# Water treatment and *E. coli* in drinking water: Household responses to (invisible) water quality risks

**DOI:** 10.1371/journal.pone.0331258

**Published:** 2026-01-23

**Authors:** Akito Kamei, Bhowmik Sujey Soori

**Affiliations:** 1 School of Government and Economics, Universidad Panamericana, Ciudad de México, México; 2 Economics Department, University of Arizona, Tucson, Arizona, United States of America; University of Limpopo, SOUTH AFRICA

## Abstract

**Background:** In 2024, an estimated 4 billion people lacked access to safely managed drinking water, with the greatest risks for vulnerable groups such as young children. The objective of this paper is to 1) document the share of households exposed to *E. coli* contamination at the water source, 2) examine whether households are more likely to treat their water when the source is *E. coli* contaminated, and 3) assess how household water treatment relates to *E. coli* levels in stored drinking water.

**Methods:** This paper analyzes nationally representative Multiple Indicator Cluster Surveys (MICS) data from 59,633 households in low- and middle-income countries across 25 countries in Sub-Saharan Africa, Latin America, and Asia, all of which conducted standardized water-quality tests both at the source and in stored household drinking water. The main analysis documents how household water-treatment behavior differs by the level of *E. coli* contamination in their water source using regression methods by controlling for the country-specific fixed effects and household characteristics.

**Results:** The study reveals that 78% of households do not treat their water, While 20% relying on sources with high *E. coli* contamination levels (>100 MPN/100 ml). Despite this risk, the presence of *E. coli* is positively associated with a higher likelihood of treating water by just 3 to 5 percentage points, depending on contamination levels, from an average baseline of 21%. The association is stronger in Latin America and weaker in Africa. Moreover, analysis of stored household drinking water reveals that a substantial share of households that report treating their water still exhibit moderate to high *E. coli* contamination in stored samples.

**Conclusion:** Households’ water-treatment behavior shows only a modest association with the invisible risk of contamination, and many households still have *E. coli* contaminated drinking water even after treating it. These results highlight the need for WASH strategies that go beyond promoting access to treatment—specifically, strategies that inform households about contamination risks and emphasize correct and consistent application to ensure safe drinking water.

## Introduction

In 2024, an estimated 4 billion people globally lacked access to safely managed drinking water, with vulnerable populations like young children, pregnant women, and those with weak immune systems facing heightened risks from contamination [1]. This challenge is particularly acute in low- and middle-income countries (LMICs), where water contamination is widespread. In Africa, the estimated number of people without safe drinking water rose from 703 million in 2015 to 766 million in 2021 [2]. Waterborne pathogens, including fecal contamination from *Escherichia coli* (*E. coli*), pose significant health risks, especially in regions where contamination persists [3].

Despite the high prevalence of contamination in water, households typically are not able to observe *E. coli* in their water, making it difficult to assess whether and how people respond to this invisible risk. While overall water-treatment rates are informative, understanding water-treatment behavior specifically among households exposed to contamination is crucial. Households that use uncontaminated water have little need to treat it, whereas those relying on contaminated sources face higher risks yet may not adjust their behavior accordingly. Similarly, households are not able to observe the contamination level in their stored drinking water after water treatment.

The objective of this paper is to examine how household water-treatment practices differ by the level of *E. coli* contamination at the water source and how these practices relate to contamination detected in stored drinking water. This paper analyzes nationally representative Multiple Indicator Cluster Surveys (MICS) from 25 countries, all of which conducted standardized water-quality tests at both the source and in stored drinking water. For the main analysis of the water treatment behavior, we conduct a linear probability model analysis on household-level data to estimate how reported water-treatment practices vary with different levels of source contamination (that is, invisible to the household) while controlling for country fixed effects and household demographic characteristics.

## Methods

### Study design, population, and setting

#### Study design and population.

To examine the relationship between source water contamination, household water treatment decisions, and contamination in stored drinking water, we analyze the MICS household survey. The MICS, implemented by UNICEF, are nationally representative household surveys designed to collect high-quality data on living standards. MICS are conducted primarily in low- and middle-income countries, using standardized sampling methods that ensure population-level representativeness at both national and sub-national levels. In addition to detailed information on demographics, health, and water and sanitation, MICS began incorporating objectively measured water-quality testing starting in Round 6.

We use MICS 6 surveys collected between 2017 and 2021, in which a rapid water quality testing module is available. The surveys were implemented by UNICEF in collaboration with national ministries and statistics offices. From an initial sample of 29 countries, we excluded Tuvalu, Kiribati, Tonga, and the Turks and Caicos Islands, where fewer than 1,000 households completed the water testing module, to ensure sufficient data for reliable country-level statistics.

In each MICS survey, a random subsample of households within each cluster was selected for water-quality testing (typically 5 households per cluster). Depending on the country, approximately 10–25% of a random subsample of households were selected for the rapid water quality testing module (Table 1). Overall, 92.6% of households completed water testing with non-missing data, and in most countries, *E. coli* data are available for over 80–90% of the selected households. While non-consent was low (N = 653), water testing was occasionally not conducted due to the household’s inability to provide a water sample, partial completion, or errors in reading the test results. A comparison of household characteristics between those who completed the water testing and those who did not shows varying patterns across countries (see online appendix in supporting document for further discussion). The final analytical sample includes 59,633 households from 25 countries.

**Table 1:** Number of household surveys and sample included in the analysis.

	Total	Selected		Not in the sample (N)			Complete	
	N	N	(%)	No consent	Water test missing	Water treatment missing	N	(%)
**Sub-Saharan Africa**								
Central African Rep	8,133	1,228	15.1	3	188	1	1,036	84.4
Chad	18,967	2,284	12	0	171	0	2,113	92.5
Gambia	7,405	1,879	25.4	14	113	1	1,751	93.2
Zimbabwe	11,091	2,138	19.3	8	136	2	1,992	93.2
Togo	7,916	1,157	14.6	0	73	2	1,082	93.5
Eswatini	4,675	1,235	26.4	8	63	4	1,160	93.9
Madagascar	17,870	3,439	19.2	6	169	4	3,260	94.8
Benin	16,790	3,809	22.7	10	142	8	3,649	95.8
Lesotho	8,847	1,376	15.6	1	43	0	1,332	96.8
Ghana	12,939	3,230	25	3	93	3	3,131	96.9
Malawi	25,419	3,208	12.6	9	78	5	3,116	97.1
DR Congo	20,792	2,783	13.4	1	76	0	2,706	97.2
Sierra Leone	15,309	1,784	11.7	1	36	4	1,743	97.7
Guinea Bissau	7,379	1,829	24.8	1	5	1	1,822	99.6
**Latin America**								
Honduras	20,669	5,111	24.7	78	1,010	2	4,021	78.7
Dominican Republic	31,488	3,125	9.9	81	500	3	2,541	81.3
Suriname	7,915	1,982	25	281	83	2	1,616	81.5
Guyana	7,072	1,670	23.6	65	206	6	1,393	83.4
Trinidad and Tobago	7,499	1,905	25.4	36	265	3	1,601	84
Fiji	5,467	1,104	20.2	1	17	0	1,086	98.4
**Asia**								
Mongolia	13,798	2,764	20	19	169	4	2,572	93.1
Nepal	12,655	2,547	20.1	1	153	12	2,381	93.5
Lao	22,287	3,360	15.1	5	107	0	3,248	96.7
Viet Nam	13,359	3,323	24.9	11	79	2	3,231	97.2
Bangladesh	61,242	6,152	10	10	90	2	6,050	98.3
**Total**	386,983	64,422	16.6	653	4,065	71	59,633	92.6

Notes: Households that did not consent to the full survey are excluded from the sample, as household characteristics are unavailable for analysis. Cases with missing water test data reflect instances where water testing was not conducted—either because the household could not provide a water sample, the test was only partially completed, or there were errors in reading the results.

In households selected for water testing, interviewers requested permission to collect water samples from two locations: (1) the source from which the household collects water, and (2) the stored drinking water within the household. Interviewers first asked for a sample of drinking water by requesting a glass of drinking water, and then asked to be shown the water source location in order to collect a sample directly from the water source. These water samples were analyzed for the presence of *E. coli*, an indicator of fecal contamination.

The MICS survey and drinking water quality module are standardized across the countries. The protocol is developed collaboratively by the Joint Monitoring Program (JMP) and the global MICS program. An international water quality trainer, with support from national experts, led the training of field teams. National experts from regulatory agencies, research labs, or ministries of health provided technical input and oversight during fieldwork through a few field visits. Field teams obtained 100 mL samples and used portable *E. coli* enumeration tests consistent with UNICEF/JMP guidance; PoC taps were flushed for around 30 seconds before sampling. Water samples were analyzed for *E. coli* using portable membrane filtration. Field teams filtered 100mL of water through a 0.45-μm filter, placed it on CompactDry EC growth media plates, and rehydrated it with 1mL of sample water.

#### Setting.

We analyze the MICS household survey conducted in 25 low and middle-income countries across Africa, Latin America, and Asia. The African countries in our sample are the Central African Republic, Chad, Zimbabwe, Gambia, Togo, Eswatini, Madagascar, Benin, Lesotho, Ghana, Malawi, DR Congo, Sierra Leone, and Guinea-Bissau. The Latin American countries we have data on are Honduras, Dominican Republic, Suriname, Guyana, Trinidad & Tobago, and Fiji. Finally, the Asian countries that we include in our analysis are Mongolia, Nepal, Laos, Vietnam, and Bangladesh. We observe variation in water-treatment behavior both across and within countries. Because MICS surveys are nationally representative and capture diverse rural and urban settings in low- and middle-income countries, they reveal meaningful differences not only across countries but also across the regions.

### Outcome variables

To examine the relationship between household water treatment and *E. coli* contamination, the paper analyzes two types of outcomes. The first is household water treatment, defined using the MICS item that records the method households report using to treat the water sample collected for testing. Note that this response comes from the MICS water-quality module question tied to the tested water, not the general water treatment practice question. The online appendix in the supporting document shows the relationship between water treatment reported for the tested water sample and general water treatment practices. Treatment rates are lower for the tested water compared to the case when households are asked about the general water treatment practice, suggesting that households may not consistently apply the treatment methods they report using in general.

We group household water treatment responses into five categories: (i) no treatment; (ii) boiling; (iii) chlorine-based products—liquid bleach/hypochlorite, NaDCC tablets (e.g., Aquatabs), or flocculant-disinfectant powders (e.g., PUR); (iv) straining/settling; and (v) other methods. We code an indicator for “any treatment” if any of the above water treatment method is reported.

The second outcome is water quality by *E. coli*. For both the source (point of collection) and stored drinking water (point of use), we classify *E. coli* counts into three categories: <1 CFU/100 mL (low risk), 1–100 (moderate risk), and >100 (high risk). From these categories, we create binary outcomes such as “any *E. coli* (>0)” and “high risk (>100).”

### Statistical approach

We begin by presenting descriptive statistics that show the share of households exposed to different levels of *E. coli* contamination at the water source, along with associated household characteristics. We then report the prevalence of different water-treatment methods used in each country. Next, we document the proportion of households facing different levels of source contamination and further decompose these shares by whether the household reports treating its water.

We then present results from linear probability models to address three questions: (i) how the probability of treating water differs across three categories of source contamination (<1, 1–100, >100 CFU/100 mL); (ii) how the relationship between contamination and treatment varies across regions; and (iii) which specific treatment methods are more likely to be used in response to contamination. All regressions include country fixed effects and basic household-level controls, including primary water source type, urban residence, and wealth quintile. Because MICS sampling weights are country-specific, we apply household weights only when presenting country-level descriptive statistics; pooled regressions across countries do not use weights.

To address concerns about heteroskedasticity and within-cluster correlation, we use cluster-robust standard errors at the Primary Sampling Unit (PSU) level. We report the estimated coefficient—interpreted as the change in probability—along with the 95% confidence interval and the p-value for testing the null hypothesis that the coefficient is equal to zero, based on the standard errors clustered at the PSU.

We use a linear probability model to estimate binary outcomes of water treatment, allowing for interpretation of results in terms of percentage point changes in probability. We additionally estimated the model using Logit to deal with potential non-linearity, Generalized Estimating Equations (GEE), and Generalized Linear Mixed Models (GLMM) specifications (under both identity and independent covariance structures) to consider random effects within the cluster, as reported in the online appendix in the supporting document. The results are nearly identical—coefficients differ only at the third decimal place, and the associated p-values remain unchanged. For ease of interpretation, we retain the linear probability model as the main specification.

Finally, we present the share of households by the level of *E. coli* contamination in their stored drinking water, along with whether the household reported treating that water. To conclude the analysis, we compare the proportion of households by contamination level in both the source water and the stored drinking water, disaggregated by water treatment method. This allows us to examine the extent to which households continue to have contaminated drinking water even after treating it.

## Results

### *E. coli* contamination at water sources and household water treatment

Table 2 shows the share of households exposed to different levels of *E. coli* contamination at the water source and the associated household characteristics. By risk of source water, 43% (25,518) face low risk, 37% (21,844) medium risk, and 21% (12,271) high risk. Overall, about 41% of the sample lives in urban areas. Among low-risk households, 55% are urban; in the medium-risk group, 35% are urban; and in the high-risk group, just 24% are urban. We see a clear wealth gradient: the poorest quintile makes up 31% of the high-risk group but only 13% of the low-risk group; by contrast, the richest quintile accounts for 10% of high-risk households and 28% of low-risk ones.

**Table 2:** Household characteristics by water source contamination level (share)

	Low risk	Moderate risk	High risk
Urban	0.55	0.35	0.24
**Socioeconomic level**			
Poorest	0.13	0.23	0.31
Poor	0.16	0.21	0.24
Middle	0.20	0.20	0.20
Rich	0.23	0.20	0.15
Richest	0.28	0.17	0.10
**Primary water source**			
Piped water	0.41	0.30	0.17
Tube/Well/Borehole	0.27	0.28	0.12
Protected well/spring	0.03	0.09	0.14
Unprotected well/spring	0.02	0.11	0.30
Surface/Rain water	0.02	0.07	0.18
Packaged/Bottled water	0.21	0.14	0.07
Others	0.03	0.01	0.01
N	25,518 (42.8%)	21,844 (36.6%)	12,271 (20.6%)

Notes: The table presents the household characteristics across 25 countries.

The types of water sources follow the risk levels. Piped water supplies 41% of low-risk households but only 17% of high-risk ones. Unprotected wells or springs increase from 2% in the low-risk group to 30% in the high-risk group. Surface or rainwater goes from 2% (low risk) to 18% (high risk). Packaged or bottled water drops from 21% in low-risk households to 7% in high-risk ones.

**Fig 1:**
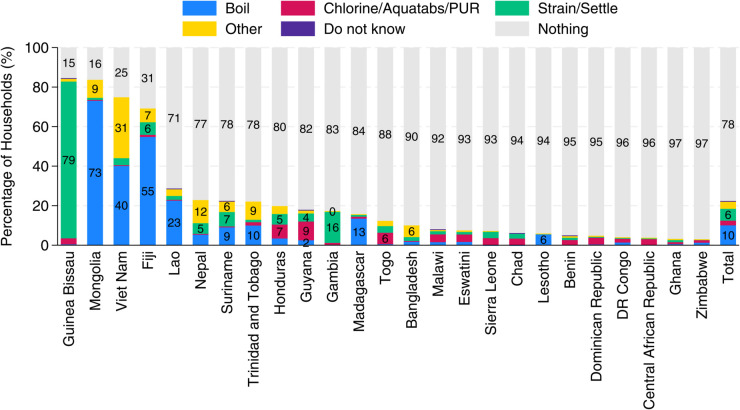
Proportion of households by water treatment method and country. This figure shows the share of households using different water treatment methods across countries. The data are weighted to be nationally representative, and the “Total” value represents the simple average of country-level estimates. “Other” methods include practices such as the use of commercially available water filters.

Fig 1 shows the prevalence of water treatment practices by country. Overall, water treatment rates are low across most countries, with 78% of the households not treating water. With the exception of Guinea-Bissau, Mongolia, and Vietnam, fewer than 25% of households report treating their water. In Guinea-Bissau, treatment is primarily done through straining and settling, while in Mongolia and Vietnam, boiling is the most commonly reported method.

**Fig 2:**
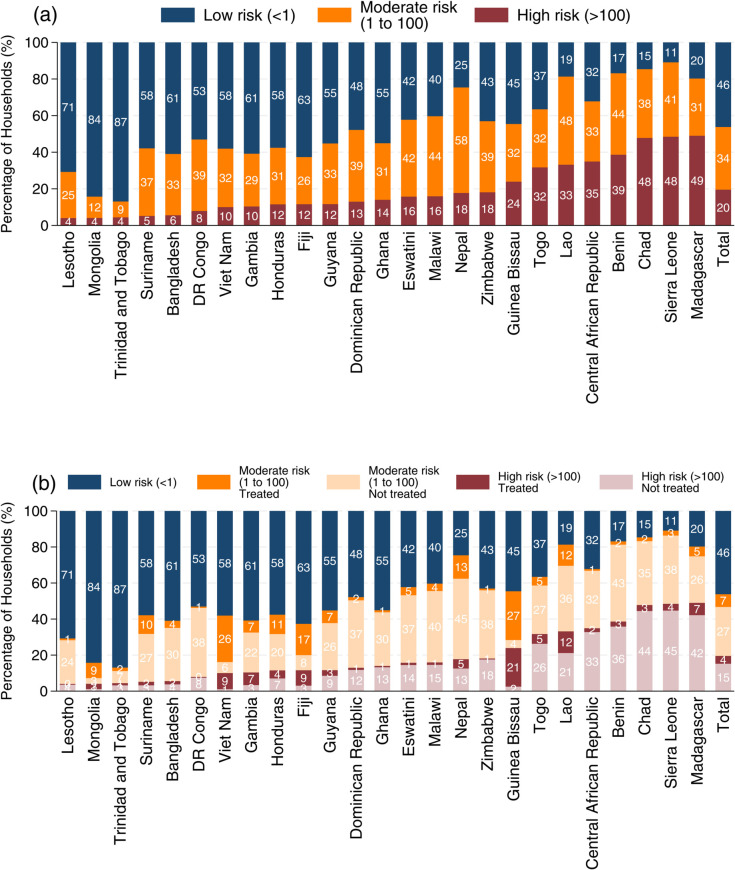
Proportion of households by water source contamination and water treatment practice across countries. (A) share of households by levels of *E. coli* contamination at the water source. (B) share of households by both source contamination and household water treatment practices. The figure illustrates cross-country variation in water quality and treatment behaviors. Data are weighted to ensure national representativeness, and the “Total” values reflect the average of country-level estimates. Water treatment methods include boiling, chlorine products, Aquatabs or PUR, straining or settling, and other methods.

Fig 2(a) reports the *E. coli* contamination levels at the water source across countries, with around 20% of the households exposed to high-risk contamination. Countries such as Chad, Sierra Leone, and Madagascar exhibit high levels of contamination (>100 CFU/100ml). These countries not only have a greater share of households exposed to high-risk water sources, but also a substantial proportion with sources with moderate *E. coli* levels (1–100 CFU/100ml), resulting in less than 20% having access to low-risk water. Lesotho, Mongolia, and Trinidad and Tobago are characterized by relatively low levels of *E. coli* contamination, with over 70% accessing low-risk sources.

While overall water treatment rates are low, the critical measure is the treatment rate among households exposed to contaminated source water. Fig 2(b) builds on panel (a) by showing, among households exposed to moderate or high contamination risk, the share that treat their water and the share that do not. Madagascar, Sierra Leone, and Chad show a large proportion of households rely on water sources with high levels of *E. coli* contamination, while the prevalence of water treatment remains low. For example, in Madagascar, 42% do not treat their water despite sourcing it from highly contaminated sources.

In Lesotho, although a relatively small share of households is exposed to moderate to high-risk water sources, overall water treatment rates are low, leading to 24% being exposed to moderate-risk water without any treatment. In contrast, countries such as Mongolia, Vietnam, and Fiji show a low proportion of households exposed to highly contaminated water without treatment, reflecting their relatively high overall treatment rates. A similar pattern is observed in Guinea-Bissau, though this is primarily driven by the common use of straining and settling as a treatment method.

### Water treatment behavior by source water contamination

Table 3 presents the empirical results on how decisions to treat water differ by the contamination level of the water source. Panel A shows that the probability of applying treatment is 3 percentage points higher when the water source has some presence of *E. coli* but less than 100 CFU compared to no presence. The probability increases by 5 percentage points if the water is highly contaminated, with the water containing more than 100 CFU.

**Table 3:** Water source *E. coli* contamination and the probability of household water treatment decision, overall (N = 59,633) and by region.

Panel A: Dependent var — Water treated (yes = 1, no = 0)									
	**(1)**								
	**Overall**								
	**Coef.**	**95% CI**	**p-value**						
Moderate risk	0.03	[0.02,0.04]	0.00						
High risk	0.05	[0.04,0.06]	0.00						
Mean	0.213								
N	59,633								
**Panel B: Dependent var — Water treated (yes = 1, no = 0) by region**									
	**(2)**			**(3)**			**(4)**		
	**Africa**			**Latin America**			**Asia**		
	**Coef.**	**95% CI**	**p-value**	**Coef.**	**95% CI**	**p-value**	**Coef.**	**95% CI**	**p-value**
Moderate risk	0.02	[0.01,0.03]	0.00	0.06	[0.04,0.07]	0.00	0.02	[0.00,0.03]	0.02
High risk	0.03	[0.01,0.04]	0.00	0.08	[0.05,0.10]	0.00	0.05	[0.02,0.07]	0.00
Mean	0.117			0.224			0.368		
N	29,893			12,258			17,482		
**Panel C: Dependent var — Boiling, Chlorine, and Strain/Settle (yes = 1, no = 0)**									
	**(5)**			**(6)**			**(7)**		
	**Boil**			**Chlorine/Aquatabs/PUR**			**Strain/Settle**		
	**Coef.**	**95% CI**	**p-value**	**Coef.**	**95% CI**	**p-value**	**Coef.**	**95% CI**	**p-value**
Moderate to high risk	–0.00	[–0.01,0.00]	0.94	0.01	[0.01,0.01]	0.00	0.02	[0.01,0.02]	0.00
Very high risk	–0.00	[–0.01,0.01]	0.70	0.02	[0.01,0.02]	0.00	0.03	[0.02,0.03]	0.00
Mean	0.101			0.022			0.051		
N	59,633			59,633			59,633		

Notes: All regressions include country fixed effects, water source type, urban/rural status, and household socioeconomic status based on an asset-based wealth index. Risk categories in the regression are defined as follows: <1 CFU/100 mL (low risk), 1–100 (moderate risk), and >100 (high risk). Robust standard errors are clustered at the primary sampling unit (PSU) level.

The presence of *E. coli* in source water is associated with a higher likelihood of household water treatment in all regions (Panel B). However, the increase is smaller in Sub-Saharan Africa. The largest effect is observed in Latin America, where *E. coli* contamination is associated with a 6 to 8 percentage point increase in water treatment.

Panel C examines how the use of specific water treatment methods varies with the level of source water contamination. No increase in treatment is observed for boiling. However, chlorination methods—including Aquatabs and PUR—as well as straining and settling, show positive and statistically significant associations with higher contamination levels.

The online appendix in the supporting document presents the same estimates as Panel A but using Logit, GEE, and GLMM specifications as robustness checks. The results are nearly identical—coefficients differ only at the third decimal place. We also present empirical results on water treatment by source type. However, because water treatment behavior and source selection involve complex decision-making processes that fall outside the scope of this paper, detailed estimations by source type are reported in the Appendix.

### Household water treatment and *E. coli* in drinking water

**Fig 3:**
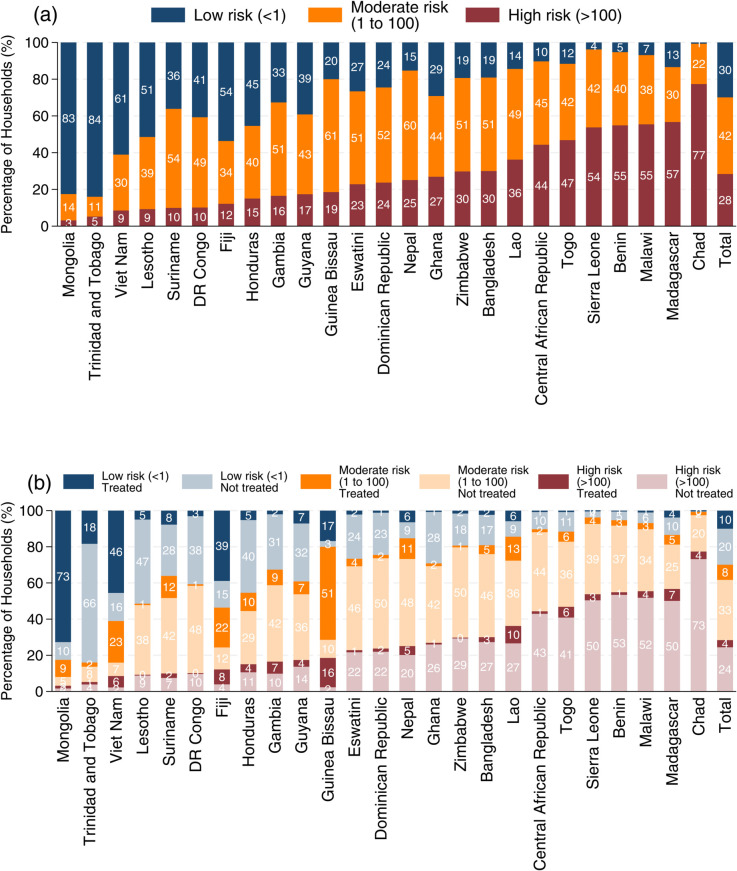
Proportion of households by stored drinking water contamination and water treatment practice across countries. (A) share of households by levels of *E. coli* contamination in stored drinking water. (B) share by both stored water contamination and household water treatment practices. Water treatment methods include boiling, chlorine products, Aquatabs or PUR, straining or settling, and other approaches. The data are weighted to ensure national representativeness, and the “Total” values represent the simple average of country-level estimates.

Fig 3(a) presents the levels of *E. coli* contamination in household stored drinking water. Compared to contamination at the source, drinking water shows higher levels of contamination. In Mongolia, 83% of households consume low-risk drinking water. In contrast, in Chad, only 1% consume low-risk drinking water, while 77% consume water with high levels of contamination.

Fig 3(b) reports a decomposition of drinking water contamination levels based on whether households treat their water. In Mongolia and Vietnam, where water treatment—particularly boiling—is widely practiced, a high share of households achieve low-risk drinking water. In contrast, in Guinea-Bissau, despite high reported treatment rates, which are primarily through straining and settling, 51% are still exposed to moderately contaminated drinking water. Additionally, 16% in Guinea-Bissau are exposed to high-risk drinking water despite treating it, suggesting limited effectiveness of the treatment methods used.

**Fig 4:**
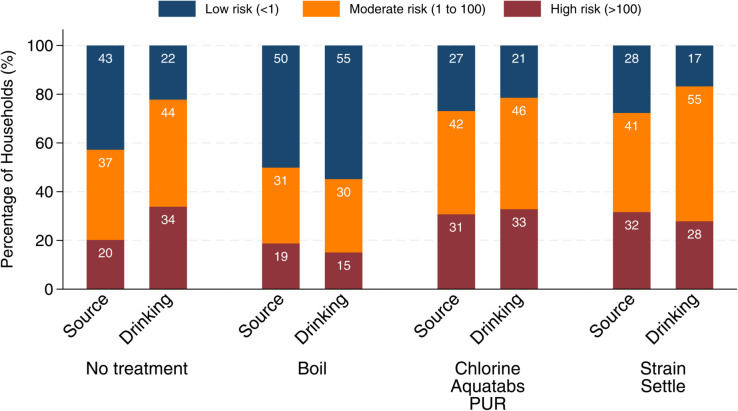
Proportion of households by water contamination levels in source and stored drinking water. This figure presents the distribution of households by levels of water contamination measured at both the source and the stored drinking water at the point of consumption.

Fig 4 presents the transition of water contamination levels between the water source and stored drinking water. For households that do not treat their water, the share of households classified as low risk drops from 43% at the source to 22% in stored drinking water, while the proportions in moderate and high-risk categories increase.

For any of the water treatment methods—boiling, chlorine-based products, or straining and settling—the overall proportion of households of contamination levels changes only modestly from source to drinking water. Among those who boil water, 19% of source samples are classified as high risk, decreasing slightly to 15% in stored drinking water. This suggests some associated reduction in contamination from boiling, but also underscores that boiling does not guarantee *E. coli*–free drinking water for all households. Similar patterns are observed for chlorine-based treatments and straining/settling.

It is important to note that these results do not imply causal effects. Households’ choice of treatment method is closely linked to initial water quality and the surrounding environment, including storage conditions. Although water treatment methods such as boiling and chlorine-based products are effective at eliminating *E. coli* under controlled conditions, water can become recontaminated during storage in real-world settings. In addition, improper application of chlorine or the use of highly turbid or heavily contaminated water may require higher doses than households typically use. While the analysis in this paper is descriptive, the results from observational data highlight that a substantial share of households continue to consume water containing *E. coli*, even when they report using treatment methods.

## Discussion

Our results show that a large share of households across LMIC are exposed to high levels of source-water contamination, yet water-treatment rates are low and do not vary substantially with the degree of *E. coli* contamination detected in source water. Other studies using MICS data similarly document high levels of *E. coli* contamination. For example, one study in Ghana found that 62% of urban households and 89% of rural households had *E. coli* contamination at the point of consumption [4]. Evidence from 27 MICS countries further shows widespread contamination, with point-of-consumption *E. coli* detected in 19% to 99% of households [5].

This paper contributes to the WASH literature by providing evidence with broad external validity—across countries and within diverse national and regional contexts. First, it documents how households’ water treatment behaviors differ in response to the invisible contamination risks. Prior work has shown that perceptions, more than objective risk, often drive household decisions [6], and access to improved water sources may even cause households to reduce water treatment efforts at home [7–9]. Since *E. coli* contamination is invisible, households may incorrectly believe their water is safe [10]. While informing households about contamination has been shown to increase treatment in some contexts [11], the effectiveness of such informational interventions varies widely depending on the design and context of the study [12,13]. *E. coli* contamination is inherently invisible to households, meaning that the treatment behaviors we observe are formed under imperfect information about actual water quality.

The study provides observational evidence on the relationship between household water treatment and drinking water quality. Although systematic reviews conclude that point-of-use water treatment is highly effective [14–16], its real-world effectiveness is often reduced due to challenges in consistent use and unsanitary storage conditions [17,18]. Randomized controlled trials in Bangladesh, Kenya, and Zimbabwe show mixed impacts of water treatment on health outcomes, emphasizing the importance of adherence [19,20]. Our findings align with this literature, showing that while treatment reduces contamination, achieving fully safe drinking water requires more than simple access to water treatment methods.

## Conclusion

Using data from 14 Sub-Saharan African countries, 6 Latin American countries, and 5 Asian countries, this paper documents *E. coli* contamination in source water and examines household responses to this invisible risk through water treatment practices. It also explores the relationship between these treatment practices and *E. coli* levels in stored drinking water.

Our analysis shows that only a small percentage of households treat their water, even when facing high contamination risks from *E. coli* in the water source, with levels exceeding 100 MPN/100ml. We find that as contamination levels increase, households are more likely to treat their water; However, the magnitude of this response is modest—ranging from 3 to 5 percentage points depending on the level of *E. coli* contamination. The response varies across regions and is primarily driven by increased use of chlorine, Aquatabs, PUR, and methods such as straining and settling, while no significant change is observed for boiling.

Access to water, sanitation, and hygiene is a fundamental human right. However, the results in this paper show that a large share of households is exposed to high levels of water contamination at the source—far from meeting the SDG targets of safe drinking water for all by 2030. Governments also cannot expect households to respond naturally to contamination levels, as the risk is essentially “invisible.” This study reveals that water treatment rates vary only slightly across contamination levels. The behavioral response to contamination is especially low in Sub-Saharan Africa, leaving many people exposed to highly contaminated water.

Compounding the problem, and regardless of causality, the analysis shows that households rarely achieve *E. coli*-free water even when they report treating it. These findings underscore the need for more robust and effective WASH interventions, particularly in regions where water treatment adoption remains persistently low. Addressing these gaps is critical for reducing waterborne diseases such as diarrhea and saving lives across the region.

Despite highlighting the link between water treatment and *E. coli* levels, the paper has several limitations. One such limitation is that we do not make any causal claims regarding the effectiveness of water treatment in reducing *E. coli* contamination. Instead, the paper focuses on the reduction of *E. coli* by leveraging the availability of objective water quality measures, which are not typically included in household surveys. Another limitation is that the MICS data rely on self-reported information for water treatment practices, which is subject to social desirability bias. Respondents may over-report their adherence to water treatment methods, potentially leading to overestimating water treatment behavior. If the bias exists, the already concerning results of low water treatment practices may be even worse than what is reported in this paper. A future study using a large-scale longitudinal water-quality monitoring system, combined with causal identification strategies and broader external validity, would provide deeper insight into households’ complex responses to invisible water risks and their water-treatment behaviors.

## Supporting information

S1 File Online Appendix. (PDF)
