# Supplementary material for: Water treatment and E. coli in drinking water: Household responses to (invisible) water quality risks
Source: PLoS One. 2026 Jan 23;21(1):e0331258. doi: 10.1371/journal.pone.0331258 (PMC12829840; doi:10.1371/journal.pone.0331258)
Supplement: S1 File — (PDF) [file pone.0331258.s001.pdf]

Supplemental Information
— For Online Publication Only —

Water Treatment and E. Coli in Drinking Water: Household Responses to (Invisible) Water Quality Risks

Akito Kamei, and Bhowmik Sujey Soori

1 Sample

Table 1 in the main text presents the number of households included in the analysis and those excluded in each country. Honduras, the Dominican Republic, and Suriname have the lowest completion rates, at 79%, 81%, and 82%, respectively.

Comparing household characteristics between those with and without water testing data across the full sample captures differences in completion rates at the country level, rather than differences within countries. For example, in Honduras, 1,088 households are missing water testing information. As a result, households with missing data in the full sample will disproportionately reflect the characteristics of Honduras, rather than revealing within-country differences.

Tables S1.1–S1.3 present household characteristics by testing completion status for Honduras, Suriname, and the Dominican Republic—the three countries with the lowest completion rates. Each country shows distinct patterns in the characteristics of households with missing water sample information. In Honduras, households with piped water are less likely to have complete data, while those using packaged or bottled water show higher completion rates. In contrast, this pattern is not observed in Suriname, where missing water sample data are more concentrated in urban areas and appear less related to the type of primary water source other than surface or rain water.

Table S1.1: Household Characteristics for In/out Final Sample (Honduras)

	In sample	Out sample	Diff	P-value
Urban	0.47	0.50	-0.03	0.12
Have under 5 children	0.33	0.32	0.01	0.38
**Socioeconomic level**				
Poorest	0.18	0.19	-0.01	0.34
Poor	0.17	0.26	-0.09	0
Middle	0.20	0.22	-0.02	0.18
Rich	0.22	0.16	0.06	0
Richest	0.23	0.17	0.06	0
**Primary water source**				
Piped water	0.38	0.63	-0.25	0
Tube/Well/Borehole	0.01	0.01	-0.01	0.03
Protected well/spring	0.02	0.01	0	0.49
Unprotected well/spring	0.02	0.02	0	0.55
Surface/Rain water	0.01	0.01	0	0.15
Packaged/Bottled water	0.51	0.26	0.25	0
Others	0.01	0.03	-0.02	0
Observations	4,021	1,090	5,111	5,111

Notes: The table presents household characteristics for two groups: those included in the analysis sample—with complete information (consent, valid water quality data from both the source and stored drinking water, and valid water treatment data)—and those excluded from the analysis sample.

Table S1.2: Household Characteristics for In/out Final Sample (Suriname)

	In sample	Out sample	Diff	P-value
Urban	0.78	0.90	-0.12	0
Have under 5 children	0.25	0.23	0.02	0.33
**Socioeconomic level**				
Poorest	0.20	0.18	0.02	0.43
Poor	0.18	0.22	-0.03	0.14
Middle	0.22	0.21	0.01	0.54
Rich	0.21	0.14	0.07	0
Richest	0.19	0.25	-0.06	0
**Primary water source**				
Piped water	0.70	0.72	-0.02	0.40
Tube/Well/Borehole	0	0	0	0.32
Protected well/spring	0.02	0.03	-0.01	0.15
Unprotected well/spring	0	0	0	0.97
Surface/Rain water	0.17	0.11	0.06	0
Packaged/Bottled water	0.09	0.11	-0.02	0.35
Others	0.01	0.02	-0.02	0
Observations	1,616	366	1,982	1,982

Notes: The table presents household characteristics for two groups: those included in the analysis sample—with complete information (consent, valid water quality data from both the source and stored drinking water, and valid water treatment data)—and those excluded from the analysis sample.

Table S1.3: Household Characteristics for In/out Final Sample (DominicanRepublic)

	In sample	Out sample	Diff	P-value
Urban	0.76	0.74	0.02	0.27
Have under 5 children	0.24	0.19	0.05	0.01
**Socioeconomic level**				
Poorest	0.22	0.26	-0.05	0.01
Poor	0.20	0.23	-0.03	0.07
Middle	0.20	0.19	0.01	0.43
Rich	0.20	0.20	0	0.94
Richest	0.19	0.12	0.06	0
**Primary water source**				
Piped water	0.07	0.06	0.01	0.33
Tube/Well/Borehole	0.01	0	0	0.16
Protected well/spring	0	0.01	-0.01	0.10
Unprotected well/spring	0	0	0	0.31
Surface/Rain water	0.02	0.03	-0.01	0.23
Packaged/Bottled water	0.81	0.62	0.19	0
Others	0.01	0.01	-0.01	0.20
Observations	2,541	584	3,125	3,125

Notes: The table presents household characteristics for two groups: those included in the analysis sample—with complete information (consent, valid water quality data from both the source and stored drinking water, and valid water treatment data)—and those excluded from the analysis sample.

2 Reported Water Treatment: General Practices vs. Tested Water Sample

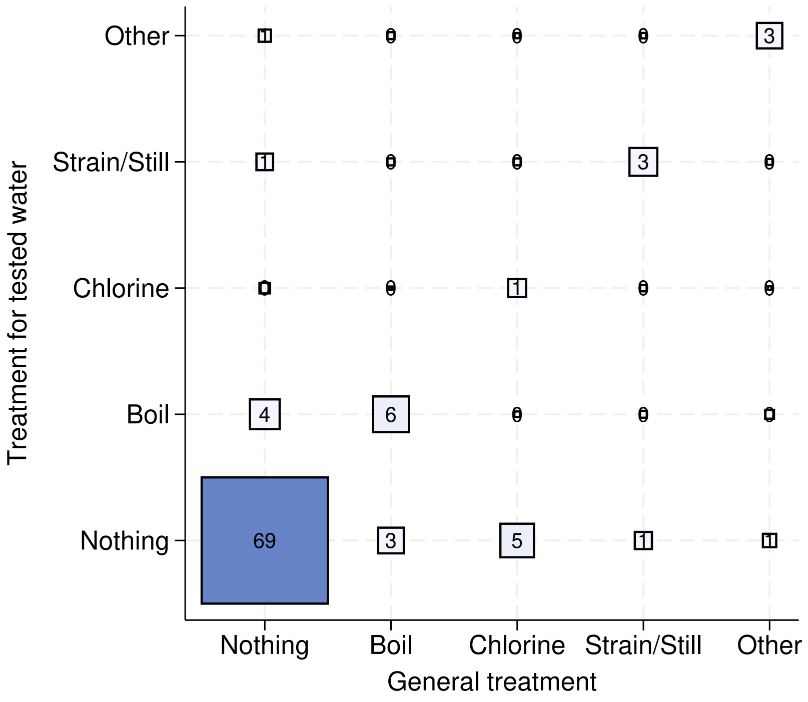

Figure S1.1: **Cross-Tabulation of Water Treatment Methods: Reported General Practices vs. Tested Water Sample (Proportion of Households).** The x-axis shows households' reported general water treatment practices, and the y-axis shows the treatment reported for the specific water sample tested in the water-quality module.

3 Alternative Methodologies (Logit, GEE, and GLMM)

The study presents results from the linear probability model for ease of interpretation. The estimates remain virtually unchanged when alternative specifications—logit, GEE (logit), and GLMM (logit)—are employed.

Table S1.1: Robustness: LPM and Marginal Effects (Logit, GEE, GLMM)

	(1) LPM			(2) Logit		
	Coef.	95% CI	p-value	Coef.	95% CI	p-value
Base: Low risk						
Moderate risk	0.03	[0.02,0.04]	0.00	0.03	[0.03,0.04]	0.00
High risk	0.05	[0.04,0.06]	0.00	0.05	[0.04,0.06]	0.00
Mean	0.21			0.21		
N	59,633			59,633		

	(1) GEE (logit)			(2) GLMM (logit)		
	Coef.	95% CI	p-value	Coef.	95% CI	p-value
Base: Low risk						
Moderate risk	0.03	[0.02,0.04]	0.00			
High risk	0.05	[0.04,0.06]	0.00			
Mean	0.21			0.21		
N	59,633			59,633		

Note: Column (1) reports LPM coefficients. Columns (2)–(4) report average marginal effects (AMEs) from logit, GEE-logit, and GLMM-logit, respectively. All specifications include country fixed effects, water source type, urban/rural status, and wealth-index controls. Robust standard errors are clustered at the primary sampling unit (PSU) level.

4 Water Treatment Response by Source Type

The empirical analysis shows higher treatment rates in response to higher levels of source contamination only for households using piped water, as well as packaged and bottled water.

Table S1.1: Water Treatment Response to Source E. coli Contamination, by Source Type

	(1) Piped/water			(2) Tube/Well/Borehole			(3) Protected well/spring		
	Coef.	95% CI	p-value	Coef.	95% CI	p-value	Coef.	95% CI	p-value
Base: Low risk									
Moderate risk	0.03	[0.02,0.05]	0.00	0.00	[-0.01,0.01]	0.73	-0.00	[-0.03,0.03]	0.89
High risk	0.08	[0.06,0.11]	0.00	0.01	[-0.01,0.03]	0.36	0.00	[-0.03,0.04]	0.87
Mean	0.28			0.13			0.39		
N	17,987			14,836			4,823		

	(4) Unprotected well/spring			(5) Surface/Rainfall			(6) Packaged/Bottle		
	Coef.	95% CI	p-value	Coef.	95% CI	p-value	Coef.	95% CI	p-value
Base: Low risk									
Moderate risk	0.00	[-0.02,0.03]	0.71	-0.02	[-0.06,0.02]	0.34	0.03	[0.02,0.04]	0.00
High risk	-0.02	[-0.04,0.01]	0.24	-0.02	[-0.06,0.02]	0.39	0.02	[0.00,0.05]	0.03
Mean	0.21			0.23			0.07		
N	7,501			4,769			8,566		

Note: All regressions include country fixed effects, urban/rural status, and household socioeconomic status based on an asset-based wealth index. Risk categories in the regression are defined as follows: $<$ 1 CFU/100 mL (low risk), 1–100 (moderate risk), and $>$ 100 (high risk). Robust standard errors are clustered at the primary sampling unit (PSU) level.
